# Chemokines and Pain in the Trigeminal System

**DOI:** 10.3389/fpain.2021.689314

**Published:** 2021-07-09

**Authors:** Oscar O. Solis-Castro, Natalie Wong, Fiona M. Boissonade

**Affiliations:** ^1^School of Clinical Dentistry, University of Sheffield, Sheffield, United Kingdom; ^2^The Neuroscience Institute, University of Sheffield, Sheffield, United Kingdom

**Keywords:** chemokines, trigeminal nerve, trigeminal ganglion, pain, neuropathic pain, inflammatory pain

## Abstract

Chemotactic cytokines or chemokines are a large family of secreted proteins able to induce chemotaxis. Chemokines are categorized according to their primary amino acid sequence, and in particular their cysteine residues that form disulphide bonds to maintain the structure: CC, CXC, CX3C, and XC, in which X represents variable amino acids. Among their many roles, chemokines are known to be key players in pain modulation in the peripheral and central nervous systems. Thus, they are promising candidates for novel therapeutics that could replace current, often ineffective treatments. The spinal and trigeminal systems are intrinsically different beyond their anatomical location, and it has been suggested that there are also differences in their sensory mechanisms. Hence, understanding the different mechanisms involved in pain modulation for each system could aid in developing appropriate pharmacological alternatives. Here, we aim to describe the current landscape of chemokines that have been studied specifically with regard to trigeminal pain. Searching PubMed and Google Scholar, we identified 30 reports describing chemokines in animal models of trigeminal pain, and 15 reports describing chemokines involved in human pain associated with the trigeminal system. This review highlights the chemokines studied to date at different levels of the trigeminal system, their cellular localization and, where available, their role in a variety of animal pain models.

## Introduction

Pain is defined as an unpleasant sensory and emotional experience associated with, or resembling that associated with, actual or potential tissue damage (1). Pain represents a major global health problem and is considered an area of significant unmet clinical need. Orofacial pain (OP) can be classified into seven categories: (i) OP attributed to disorders of dentoalveolar and anatomically related structures, (ii) myofascial OP, (iii) temporomandibular joint pain, (iv) OP attributed to lesion or disease of the cranial nerves, (v) OP resembling presentations of primary headaches, (vi) idiopathic OP, and (vii) psychosocial (2).

Anatomically, orofacial pain originates from the trigeminal nerve (cranial nerve V), which innervates facial, oral and nasal structures, and the meninges surrounding the brain. The peripheral branches of the trigeminal nerve merge to form the ophthalmic, maxillary, and mandibular nerve divisions. Centrally, most trigeminal sensory A-delta and C-fibres enter the pons and descend to form the spinal trigeminal tract, which extends caudally into the upper cervical cord with terminals synapsing on neurons of the spinal trigeminal nucleus, located medially to the spinal trigeminal tract. The afferent fibres respond to a range of thermal, mechanical, and chemical nociceptive stimuli (3).

The cellular and molecular mechanisms of pain involve an interplay between neurons, glial and other non-neuronal cells, triggering reactions that induce and maintain altered neuronal excitability linked with chronic pain. Among numerous mediators in these interactions, chemokine or chemotactic cytokines have been shown to have a pivotal role in pain modulation (4). Chemokines are a robust family of secreted proteins that signal through G protein-coupled receptors (chemokine receptors). They are categorized according to their primary amino acid sequence, and in particular their cysteine residues that form disulphide bonds to maintain the structure: CC, CXC, CX3C, and XC, in which X represents variable amino acids (5).

Comprehensive reviews of chemokines as potential therapeutic targets for chronic pain have been published elsewhere (4, 6). Nonetheless, most of the literature available is focused on the spinal system and relatively few papers include the role of chemokines in the trigeminal system. Despite neuropathic pain being observed more frequently in the trigeminal system than in spinal systems (7–9), many more studies have focussed on spinal systems (7–9). Additionally, the pathophysiology of the trigeminal nerve and spinal nerves are clearly different (10), and recent transcriptomic and translational profiling evidence further supports different molecular mechanisms underlying spinal and trigeminal neuropathies and pain processing (11, 12).

The aim of the present review is not to directly compare and contrast the trigeminal and the spinal systems. Rather, our objective is to review and summarise the current understanding of the potential role of chemokines in the neurobiology of trigeminal pain.

## Search Summary

We explored results returned by searching PubMed and Google Scholar using the terms “chemokines AND trigeminal pain,” “chemokines AND migraine,” and “chemokines AND trigeminal neuralgia,” as well as permutations of “each chemokine” (e.g., CCL2, CX3CL1, etc.), AND the terms “Trigeminal Pain” or “TMJ” or “Tooth Pain” or “Dental pain” or “Trigeminal Ganglion” or “Trigeminal Pain.”

**Figure 1:**
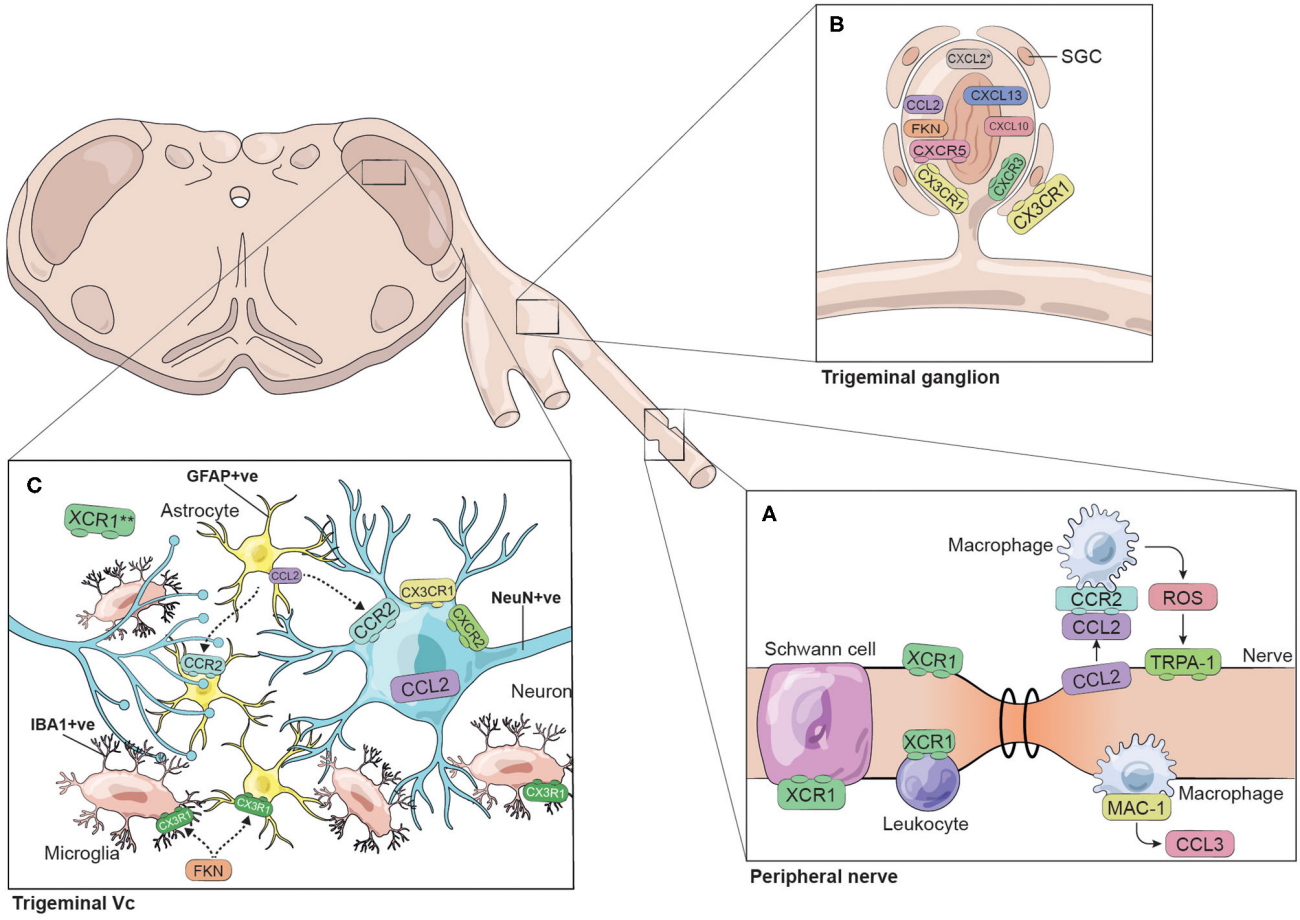
Chemokines and chemokine receptors identified in the trigeminal system. The figure represents the chemokines and chemokine receptors identified in different trigeminal models of pain. The trigeminal system is illustrated as three regions: **(A)** peripheral nerve, **(B)** trigeminal ganglion and **(C)** trigeminal subnucleus caudalis (Vc). This simplified summary does not necessarily consider time-dependent responses nor specific interactions between cellular players. *CXCL2 was identified in cultured TG cells. **XCR1 has been identified in the trigeminal Vc colocalizing with vGlut2-containing terminals.

We identified 30 reports of different animal models where pain was studied in the trigeminal system and that mentioned directly or indirectly at least one chemokine (Table 1). We found 15 reports exploring chemokine levels in human samples associated with migraine, tension-type headache, trigeminal neuralgia, pulpitis, orthodontic pain, or hemifacial spasm (Table 2). We excluded reports in which pain was not part of the research question. A simplified illustrative summary of the findings can be seen in Figure 1.

**Table 1:** Summary of chemokines reported in animal models of trigeminal pain.

**Chemokine(s)**	**Injury model**	**Animal**	**Region**	**Timing**	**IHC co-localization**	**Behavioural test**	**Effect**	**Reference**
CCL2	DED	Mouse	TG	Day 21	Not explored	Eye closing ratio, *ex vivo* corneal sensitivity	Associated with nociception	Fakih et al. (13)
CCL2	IONC	Mouse	PN	Day 10	Not explored	Head withdrawal	Pronociceptive	Trevisan et al. (14)
CCL2	DED	Mouse	BS	Day 7	Not explored	Eye wiping and reduced weight gain	Associated with nociception	Launay et al. (15)
CCL2/CCR2	IONL	Rat	TG	Day 14	CCL2 -IB4; -CGRP; -SP	Head withdrawal	Pronociceptive	Dauvergne et al. (16)
CCL2/CCR2	IONL	Rat	BS	Day 14	CCL2-GFAP	Head withdrawal	Pronociceptive	
CCL2/CCR2	IONL	Rat	BS	Day 1	CCL2-NeuN; CCR2-GFAP	Head withdrawal	Associated with nociception	Kubíčková et al. (7)
CCL2/CCR2	IONL	Rat	BS	Day 3	CCL2-GFAP; CCR2-GFAP	Head withdrawal	Associated with nociception	
CCL2/CCR2	Tooth injury	Rat	BS	Days 3 and 14	CCL2-GFAP; CCR2-NeuN	Head withdrawal	Pronociceptive	Luo et al. (17)
CCL2/CCR2	Transgenic – TNFα	Mouse	TG	Not explored	Not explored	Drinking (temperature/pressure)	Associated with nociception	Rozas et al. (18)
CCL2/CCR2	CFA	Rat	TG	Day 2	Small- and medium-sized neurons	Head withdrawal	Associated with nociception	Takeda et al. (19)
CCL2/CCR2	Tooth injury	Rat	TG	Day 3	Small- and medium-sized neurons	Face grooming	Associated with nociception	Yang et al. (20)
CCL2/CCR2	IAMNT	Mouse	BS	Days 3–21	CCL2-GFAP; CCR2-NeuN	Heat (head withdrawal)	Pronociceptive	Zhang et al. (21)
CCL3	Mental nerve ligation	Rat	PN	Days 3–28	CCL3-MAC-1	Head withdrawal	Associated with nociception	Lee and Zhang (22)
CCL4/CCR2	Masseter muscle tendon	Rat	BS	Week 8	Not explored	Head withdrawal	Anti-hyperalgesia	Guo et al. (23)
CCL5, CCL7, CXCL9 and CXCL10 (indirect)	pIONT	Mouse	TG	Days 3–21	Not explored	Head withdrawal	Associated with nociception	Jiang et al. (24)
CCR2	IONC	Rat	TG	Days 1, 4, and 21	Not explored	Not explored (mRNA)	Associated with nociception	Korczeniewska et al. (12)
CX3CR1	CFA	Rat	BS	Day 4	CX3CR1-IBA1	Head withdrawal	Pronociceptive	Kiyomoto et al. (25)
CX3CR1/FKN	CFA	Rat	TG	Day 4	CX3CR1 neurons and SGC	*In vivo* electrophysiology	Pronociceptive	Cairns et al. (26)
CX3CR1/FKN	CFA	Rat	TG	Day 4	FKN neurons	*In vivo* electrophysiology	Pronociceptive	
CXCL1-CXCR2	Masseter muscle tendon	Rat	BS	Week 8	CXCR2-NeuN	Head withdrawal	Anti-hyperalgesia	Guo et al. (23)
CXCL10/CXCR3	pIONL	Mouse	TG	Days 3 and 10	CXCL10-TUBIII; CXCR3-TUBIII	Head withdrawal	Pronociceptive	Ju et al. (27)
CCL2/CCR2	IONL	Mouse	TG	Days 3–21	CCL2-TUBIII; CCR2-TUBIII	Drinking reward	Pronociceptive	Zhang et al. (28)
CXCL13/CXCR5	IONL	Mouse	TG	Day 10	Not explored	Head withdrawal/drinking/rota-rod	Pronociceptive	Zhang et al. (29)
CXCL2	Jaw opening	Rat	TG	Day 3	Not explored	Head withdrawal	Associated with nociception	Hawkins and Durham (30)
CXCL2	IONC	Rat	TG	Day 1	Not explored	Head withdrawal	Pronociceptive	Iwasa et al. (31)
CXCL2	CFA	Rat	TG	Not relevant (*in vitro*)	Not explored	Not explored	Associated with nociception	Chung et al. (32)
CXCL2, CCL5	Jaw opening	Rat	TG	Day 14	Not explored	Head withdrawal	Associated with nociception	Hawkins and Durham (30)
CXCL2, CXCL3, CCL5	Jaw opening	Rat	BS	Day 14	Not explored	Head withdrawal	Associated with nociception	
CXCL2, CXCL3, FKN, CCL5	Jaw opening	Rat	BS	2 h	Not explored	Head withdrawal	Associated with nociception	
CXCL3, FKN	Jaw opening	Rat	TG	2 h	Not explored	Head withdrawal	Associated with nociception	
FKN	Jaw opening	Rat	BS	Day 7	Not explored	Head withdrawal	Associated with nociception	
CXCR2	*In vitro*	Mouse	TG	Not relevant (*in vitro*)	Not explored	Not explored	Associated with nociception	Michot et al. (33)
FKN	mBSA/CFA	Rat	BS	Days 7 and 14	Not explored	Face rubbing and head flinches	Pronociceptive	Bonfante et al. (34)
FKN	mBSA/CFA	Rat	BS	24 h	Not explored	Not explored	Associated with nociception	Manuel Muñoz-Lora et al. (35)
FKN/CX3CR1	STZ	Rat	BS	Day 28	Not explored	Face rubbing	Not pronociceptive	Rocha-Neto et al. (36)
FKN/CX3CR1	CFA	Rat	BS	Days 1–5	CX3CR1-NeuN; CX3CR1-GFAP	Algometer (pressure)	Pronociceptive	Wang et al. (37)
CCL2	TNFR1/R2 -/- KO CFA	Mouse	BS	Day 14	Not Explored	Head withdrawal	Associated with nociception	McIlwrath et al. (38)
CXCL10	TNFR1/R2 -/- KO CFA	Mouse	BS	Day 14	Not Explored	Head withdrawal	Associated with nociception	
CCL5	TNFR1/R2 -/- KO CFA	Mouse	BS	Day 14	Not Explored	Head withdrawal	Associated with nociception	
CXCL9	TNFR1/R2 -/- KO CFA	Mouse	BS	Day 14	Not Explored	Head withdrawal	Associated with nociception	
XCR1	Mental nerve ligation	Rat	PN	Day 3	XCR1-TUBIII; XCR1-S100β; XCR1-CD45	*In vitro* electrophysiology	Associated with nociception	Bird et al. (39)
XCR1	Mental nerve ligation	Rat	BS	Day 3	XCR1-VGlut2	*In vitro* electrophysiology	Associated with nociception	

*IAMNT, inferior alveolar and mental nerve transection; IONC, infraorbital nerve constriction; IONL, infraorbital nerve ligation; TG, trigeminal ganglion; BS, brainstem; PN, peripheral nerve; TNF, tumour necrosis factor; XCR1, lymphotactin receptor*.

**Table 2:** Summary of chemokines reported in samples from human subjects/patients with trigeminal pain.

**Chemokine(s)**	**Condition**	**Source of sample**	**Association with pain**	**Reference**
CCL2	ID or OA	TMJ synovial fluid	Not significant	Ogura et al. (40)
CCL2	Migraine	CSF	Yes	Bø et al. (41)
CCL2	Orthodontic pain	GCF	Yes	Alikhani et al. (42)
CCL2	Third molar extraction	Adjacent tissue	Yes	Wang et al. (43)
CCL3/CCL5	Migraine	Serum	Yes	Domingues et al. (44)
CCL3/CCL8	Migraine	Serum	Yes	Duarte et al. (45)
CCL5	FDOJ	Necrotic jawbone	No	Lechner et al. (46)
CCL5	TN	Necrotic jawbone	Unknown	Lechner and von Baehr (47)
CCL5 (RANTES)	Migraine	Serum	Yes	Fidan et al. (48)
CX3CR1	Migraine	Serum	Yes	Combadière et al. (49)
CXCL8	Acute pulpitis	GCF	Unknown	Karapanou et al. (50)
CXCL8	Acute pulpitis	Pulp tissue	No	Evangelin et al. (51)
CXCL8	Acute pulpitis	Plup blood	No	Akbal Dincer et al. (52)
CXCL8	Migraine	Serum	Yes	Sarchielli et al. (53)
CXCL8	TN and HFS	Serum	No	Liu et al. (54)

*ID, internal derangement; OA, osteoarthritis; CSF, cerebrospinal fluid; GCF, gingival crevicular fluid; TN, trigeminal neuralgia; HFS, hemifacial spasm; FDOJ, fatty-degenerative osteolysis and osteonecrosis of the jawbone*.

## Animal Models of Trigeminal Pain

A number of animal models of trigeminal pain have been developed that aim to mimic the clinical conditions leading to the development of chronic orofacial pain. The search used here identified trigeminal pain models of neuropathy, inflammation, mechanical damage, and migraine that had been used specifically to investigate chemokines in trigeminal pain. The models are described in more detail below.

### Peripheral Nerve Injury (Neuropathic Pain)

Neuropathic pain is defined as pain caused by a lesion or disease of the somatosensory nervous system (55). Our search retrieved animal models of trigeminal neuropathic pain that utilised ligation (tight constriction), constriction (loose constriction), or transection (complete separation) of peripheral nerves to induce injury. The injury models that have specifically been used to study chemokines in trigeminal pain are: infraorbital nerve ligation (IONL), infraorbital nerve constriction (IONC), partial infraorbital nerve transection (pIONT), inferior alveolar and mental nerve transection (IAMNT), and mental nerve constriction (21, 29, 31, 39).

### Inflammation by Complete Freund's Adjuvant

Complete Freund's adjuvant has been widely used in the literature to promote inflammation and is commonly used in models of arthritis. In the orofacial region and in relation to chemokines, CFA has been used to study direct and indirect inflammatory pain mechanisms, either by direct injection into the temporomandibular joint (TMJ), or by injection into muscles adjacent to regions innervated by the trigeminal nerves (19, 26, 32).

### Mechanical Damage

A few models have studied the role of chemokines in damage and trigeminal pain behaviours induced *via* mechanical stimulation in the orofacial region. These models include tooth damage and prolonged jaw opening, as opposed to injection of inflammatory molecules such as CFA. It is argued that these models in particular mimic more closely orofacial pain conditions caused by mechanical damage and injury in man (20, 30).

### Streptozotocin-Induced Diabetes

STZ is a broad-spectrum antibiotic that induces toxicity and death of insulin-producing β cells (56). STZ is used to induce a diabetes model, which has been used to study diabetic neuropathy including nociception and chemokine expression (57).

### Dry-Eye Disease

Dry eye is a multifactorial health problem linked with a variety of symptoms, including ocular pain that can be reported as transient or chronic. The cornea of the eye is innervated by branches of the ophthalmic division of the trigeminal nerve (V1). Nociception related to dry eye disease can arise from insults that include infection, inflammation, trauma, adverse environmental conditions, ocular anatomy, or high tear osmolarity (58). The aetiology of dry eye disease is divided in two categories: aqueous deficiency and evaporative. Both are modelled by removing or damaging the lacrimal gland or by applying substances that promote inflammation and decrease spontaneous tear production (15).

### Transgenic Animals

Knockout mice with deletions in ion channels and inflammatory molecules involved in trigeminal pain have been used to determine the role of chemokines in pain (18, 59).

## Direct Evidence of Chemokines in Trigeminal Pain

As discussed elsewhere, chemokines and their receptors play roles in several biological processes that go beyond chemoattraction; these include roles in inflammatory responses and homeostatic functions (60). Accordingly, chemokines play a role in the nervous system in a range of different pathological conditions and physiological functions (61), including a role in pain arising in the trigeminal system. In the next section, we present relevant literature that shows the direct involvement of a particular chemokine in the modulation of pain; i.e., evidence that strongly supports a causal role in nociception rather than a general inflammatory response to injury.

### CCL2/CCR2

Chemokine ligand 2 (CCL2), or monocyte chemoattractant protein-1 (MCP-1), is a member of the CC chemokine family and binds to its receptor CCR2. It is a proinflammatory chemokine that recruits monocytes, memory T cells and dendritic cells to the sites of inflammation (21) and is also known to be a glial cell mediator (62).

CCL2/CCR2 is the most widely studied chemokine axis in relation to trigeminal pain. In comparison with other chemokines, this axis has been investigated in most of the models described in the literature discussed in the present review. These include models of neuropathic pain (IONC, IAMNT), inflammatory pain (CFA), mechanical damage, and use of transgenic animals. The CCL2/CCR2 axis has also been studied using human tissue and *in vitro* experiments. Altogether, these models have helped to identify the potential cellular origin of CCL2 and CCR2 in the trigeminal system and the pronociceptive role of this axis (14, 16, 19–21, 38, 62, 63).

CCL2 expression is upregulated in the mouse infraorbital nerve after IONC (at day 10) as observed by immunohistochemistry (IHC) (14). Although, no co-staining was done, CCL2 levels in the injured nerve remained similarly expressed even after macrophage depletion with liposome-encapsulated clodronate, indicating CCL2 can originate at least partially from other cellular components within the nerve (14). Furthermore, intraperitoneal administration of CCL2 neutralizing antibody was associated with inhibition of macrophage infiltration, a reduction of reactive oxygen species (ROS), and the inhibition of nociceptive behaviour (mechanical allodynia and cold hypersensitivity). Using data from a TRPA1 knockout mouse model, Trevisan et al. proposed the following CCL2- and TRPA1-mediated pain mechanism: upon injury, CCL2 secreted from cells within the nerve attracts CCR2-positive macrophages to the injury site; once at the injury site, macrophages generate ROS that in turn activate TRPA1 in the injured nerve (14) (Figure 1A).

At the level of the trigeminal ganglion, CCL2 has been found to be co-expressed in IB4-, CGRP- and SP-positive neurons in rats at day 14 after IONC (16). Additionally, in mechanical (tooth movement) and whisker pad CFA injection pain models, CCL2 and CCR2 were expressed to a higher degree in small- and medium-sized neurons in comparison with uninjured/sham control groups (19, 20). CCL2 and CCR2 were significantly upregulated from day 3 after tooth injury and at day 2 after CFA injection (19) (Figure 1B).

*In vitro*, dissociated trigeminal ganglion neurons from CFA-inflamed rats were found to have increased excitability, with a lower threshold current for action potential generation and a higher spike count at threshold (19). The addition of CCL2 to trigeminal ganglion (TG) cultures from CFA-treated animals further increased the spike number in comparison with cultures from naïve animals in the presence of CCL2, thus providing evidence that CFA-induced increases in TG neuron excitability may involve the CCL2/CCR2 axis (19).

In addition to the work describing CCL2/CCR2 in peripheral nerve and trigeminal ganglion neurons, several studies have also investigated CCL2 within the spinal trigeminal nucleus caudalis (Vc) in mouse and rat:

In mouse, CCL2 has been found to be upregulated in the Vc from day 3 to 21 following IAMNT, and colocalizes with GFAP-positive astrocytes as evaluated by IHC (21). Additionally, CCR2 expression has been reported in NeuN-positive neurons in mice following IAMNT, suggesting a possible interplay between CCL2-positive astrocytes and CCR2-positive neurons (21).

Findings from rat IONL nerve injury models are consistent with those from mouse models, showing increased expression of CCL2 in GFAP-positive astrocytes in nerve-injured animals (7, 16). CCL2 upregulation and astrocyte co-localization in the brainstem were also observed in a tooth movement model in rats. In this model, CCL2 expression was significantly higher than in sham animals at day 3 and 14 post-insult. Additionally, CCR2 was found in NeuN-positive neurons following IONL (17) (Figure 1C).

Contrary to results mentioned above (17, 21), contrasting evidence from a rat IONL study does not support the findings that CCR2 is expressed in neurons, but suggests CCR2 is expressed only in GFAP-positive astrocytes (7). These authors suggest that shortly following injury (day 1), CCL2 expression is triggered in the neurons, which may signal to GFAP-positive astrocytes through CCR2. By day 3, CCL2 stops being produced by Vc neurons and starts being produced in astrocytes, creating the opportunity for autocrine activity in astrocytes expressing both CCL2 and CCR2 (7). We can speculate that discrepancies in CCR2 localization could be due to slight differences in the experimental models (transection/tooth movement vs. ligation) and/or slight differences in the timing of the experimental procedures.

The pronociceptive role of CCL2 and CCR2 in neuropathic, mechanical and inflammatory-induced pain at the different levels of the trigeminal system is strongly supported by their correlation with pain-like behaviours. For instance, intracisternal administration of CCL2 in rat significantly increased face grooming activity and mechanical hypersensitivity (indicated by a withdrawal response to facial stimulation with von Frey filaments) (16). These pain-like behaviours were prevented by the intracisternal administration of the CCR2 antagonist INCB3344. Furthermore, CCR2 inhibition with INCB3344 was also shown to prevent mechanical hypersensitivity induced by ION chronic constriction injury (ION-CCI). Interestingly, it was found that CCR2 inhibition beginning 1 day before nerve ligation and followed by consecutive administration for the first 3 days after surgery, was able to delay mechanical hypersensitivity until day 8 post injury, whereas the same INCB3344 regimen, starting from day 14 post injury, was ineffective in attenuating the established mechanical allodynia. Thus, suggesting that the pronociceptive role of CCL2/CCR2 in pain modulation is restricted to the early stages following the injury (16). Similarly, in a rat model of tooth injury, delivery of CCL2 neutralizing antibody or CCR2 antagonist into the medullary region significantly decreased the development of nocifensive behaviour (face grooming) (20). Interestingly, in this tooth injury model, increased CCL2/CCR2 expression in the TG peaked at day 5 after injury and resolved by day 7, also suggesting a role of this axis in the early events of pain modulation (20). An independent tooth injury model also showed that medullary injection of CCR2 antagonist could attenuate the nocifensive behaviours on day 1 after injury (17).

Lastly, studies in mice have demonstrated that IAMNT-induced heat hyperalgesia was effectively attenuated by intracisternal injection of the CCR2 antagonist RS504393 (21).

Taken together, this evidence strongly supports the pronociceptive effect of the chemokine axis observed by the link between CCL2/CCR2 expression and behavioural changes in trigeminal pain models. Data obtained *in vitro* from the TG indicates that application of CCL2 can increase c-fos expression, suggesting a possible modulation through the c-fos pathway (20). *In vivo*, intracisternal injection of CCL2 in naïve animals increased levels of IL-1β, ITGAM, GFAP and IL-6 mRNA in the TG, suggesting an effect *via* inflammatory mediators (16).

### CX3CL1 (Fractalkine; FKN)/CX3CR1

C-X3-C motif chemokine ligand 1 (CX3CL1), or fractalkine (FKN), belongs to the CX3C chemokine family and signals only through its receptor CX3CR1, contrary to other promiscuous chemokines. FKN is the only member of the CX3C subfamily of chemokines, and its involvement in chronic pain has been of interest as a therapeutic target (64, 65). Indeed, recent reviews on the topic of FKN and chronic pain discuss the advantages of using a non-promiscuous axis as a specific therapeutic target, supported by convincing preclinical evidence of the axis' role in animal models of pain. The preclinical data encourages the exploration of clinical conditions in patients, to support the axis' translation into clinical benefits (65, 66).

Although highly studied and more understood in the spinal system, the FKN/CX3CR1 axis has been less studied in the trigeminal system. The available literature on this chemokine axis in the trigeminal system derives mostly from rat inflammatory pain models that utilise CFA injection and a STZ-induced diabetes model as described below.

In rat Vc, CX3CR1 expression has been demonstrated in astrocytes and neurons by IHC following CFA treatment, as reported by Wang et al. (37). CFA injection into the temporomandibular joint (TMJ) increased CX3CR1 and GFAP protein expression as determined by western blot (WB) when compared with sham animals. This increased protein expression was apparent from day 1 and continued until day 5 following injection, when the experiment terminated (37). These findings suggest a possible role for astrocytes in CX3CR1-mediated effects in Vc.

In a separate model of ectopic facial pain, CFA was injected into the trapezius muscle as an ectopic site, while mechanical allodynia in the facial skin was studied (25). In this model, FKN protein was elevated in Vc at day 4 post CFA injection in comparison with sham animals, as judged by WB. In relation to receptor localisation, Kiyomoto et al. (25) report somewhat contrasting findings to those presented by Wang et al. (37). Kiyomoto et al. (25) found that CX3CR1 was not expressed in astrocytes or neurons, but was present in IBA1-positive microglia (Figure 1C). This discrepancy may derive from the substantial difference in experimental models. The CX3CR1 expression did not change significantly upon inflammation as measured by WB band intensity. Notably, intracisternal administration of FKN resulted in microglial activation, which was prevented by intracisternal administration of CX3CR1 neutralizing antibody. In addition, it was found that FKN could activate IBA1-positive microglia through CX3CR1, followed by p38 phosphorylation and IL-1β secretion. Hence, it is suggested that the role of microglia in pain modulation is at least partially mediated by the FKN–CX3CR1 axis in the trigeminal system (25). In summary, the available data indicates that glial cells in rat Vc can respond to FKN through the expression of its receptor CX3CR1 (25, 37). Further research is required to clarify the localization of CX3CR1 in Vc and to determine whether the differences found in the above studies could be dependent on the differences in the research models used; for instance, exploring whether the cellular response (microglia vs. astroglia) might be related to a different mechanism of sensitization occurring after a local insult (TMJ) compared with adjacent injury sites (trapezius muscle).

Inhibition or activation of this axis was also correlated with pain behaviours. *In vivo* electrophysiological studies, allowing recording from trigeminal ganglion neurons and intra-ganglionic injection, revealed that both intra-ganglionic FKN and saline injection reduced the mechanical threshold of nociceptors supplying the temporalis muscle (defined as the minimum force required to generate a discharge in the ganglion neurons). However, FKN injection produced significantly greater mechanical sensitisation than saline injection, thus indicating a direct role in nociception. Pooled data from both sexes showed a significant reduction in the mechanical threshold after intra-ganglionic FKN injection, however the reduction in mechanical threshold lasted significantly longer in female rats (26). Sex-dependent differences in pain perception are discussed below.

Independent studies showed that intracisternal administration of FKN alone resulted in mechanical hyperalgesia (37) and mechanical allodynia with activation of IBA1-positive microglia (25). Furthermore, astrocyte inhibition by intracisternal administration of fluorocitrate showed an attenuation of CFA-induced hyperalgesia (37), and direct blocking of CX3CR1 by intracisternal administration of a neutralizing antibody was able to reverse ectopic facial skin allodynia in CFA-treated animals (25). In a separate study (67), the analgesic effect of photobiomodulation treatment was tested in CFA-treated male rats on day 7. The reduction of mechanical hyperalgesia by photobiomodulation was associated with a significant reduction in FKN immunoreactivity in the TG when compared with that in CFA-treated rats with no photobiomodulation treatment (67), indicating that FKN is elevated in the TG of animals suffering pain and further supporting its direct role in pain modulation.

It would be valuable to identify FKN localization with further precision in the TG and particularly in the trigeminal nucleus caudalis. Furthermore, it would be interesting to compare whether the FKN axis could have a similar role in pain modulation in a model of trigeminal neuropathic pain.

### CXCL13/CXCR5

C-X-C motif chemokine ligand 13 (CXCL13) is a member of the CXC chemokine family; its effects are primarily mediated by its receptor CXCR5. It is a chemoattractant for B cells and a subset of T cells, and is also involved in B-cell homing and development in lymphoid tissues (68).

CXCL13 in the trigeminal ganglion has been studied in a mouse IONL model. The ligand and its receptor CXCR5 have both been located in class III β-tubulin (TUBIII)-expressing ganglion cells (Figure 1B). Additionally, RNA and protein expression (as assessed by RT-qPCR and WB, respectively) for both the ligand and receptor are reported to be increased in mouse trigeminal ganglion following IONL in IONL-injured mice when compared with sham and naïve animals (28). The use of a CXCR5 -/- knock out (KO) strain allowed the determination of the role of the axis in pain and its possible mechanism of action. The KO mice showed less IONL-induced mechanical allodynia in comparison with wild-type (WT) animals and did not respond to exogenous CXCL13 injection. CXCL13 knock down by shRNA also reduced IONL-induced mechanical allodynia, while WT mice treated with intra-ganglionic injection of CXCL13 had an exacerbated sensitivity to mechanical stimuli. Interestingly, comparing phosphorylated vs. non-phosphorylated forms by WB, it was found that the CXCR5/CXCL13 chemokine axis could be acting through the phosphorylation of p38 and ERK but not JNK to mediate the expression of pro-inflammatory cytokines IL-1β and TNFα in trigeminal pain (28, 29). Altogether, the data strongly support the pronociceptive role of the CXCL13/CXCR5 axis in models of neuropathic pain.

### CXCL2

Chemokine C-X-C motif ligand 2 (CXCL2), also known as macrophage inflammatory protein 2 (MIP-2), is a small cytokine belonging to the CXC chemokine family. CXCL2 plays a role in recruiting neutrophils to sites of inflammation or injury (69–71).

In the IONC model of neuropathic pain, a chemokine/cytokine protein array was used to detect the levels of these molecules at different times after injury (1 day, 7 and 14 days) in TG tissue lysates. Although different chemokines were upregulated or downregulated at different time points, CXCL2, which was significantly upregulated only at day 1, was selected for further investigation. Inhibition of CXCL2 resulted in the attenuation of mechanical allodynia to levels similar to sham animals as measured by head withdrawal threshold using von Frey filaments (31). Overall, this initial evidence supports a direct role for CXCL2 in trigeminal pain (31, 32).

### XCL1/XCR1

XCL1, or lymphotactin, is a member of the XC chemokine family, and its actions are mediated by its only receptor XCR1. It is produced by natural killer cells and subsets of T cells during inflammatory and infectious responses and is a chemoattractant for T lymphocytes (39, 72).

Lymphotactin or XCL1 is the ligand of XCR1. In a rat model of mental nerve constriction, the expression of XCR1 at the injury site was found to be significantly higher in injured animals in comparison with nerves from sham operated animals at day 3 post-injury (39). XCR1 expression at the injury site was not significantly higher at day 11, suggesting its proposed pronociceptive effect might occur early after injury. XCR1 in the injured peripheral nerve was found co-localized with TUBIII nerve fibres, S100β Schwann cells and CD45-positive cells (leukocyte common antigen; Figure 1A). At the brainstem level, XCR1 was expressed in vesicular glutamate transporter 2 (VGlut2)-containing terminals in Vc (Figure 1C). Application of XCL1 to brainstem slices *in vitro* activated c-Fos, ERK and p38 in neurons in the superficial layers of Vc, and enhanced the levels of intrinsic excitability in Vc. These effects could be inhibited by blocking XCR1 with an antagonist. This study identified a novel role for XCL1/XCR1 in nociceptive processing and suggests a pronociceptive role for the XCL1/XCR1 axis in peripheral nerve injury and neuropathic pain (39).

### CXCL10/CXCR3

CXCL10 is a chemokine initially identified as interferon-γ-inducible protein 10 (IP-10), which is secreted by a number of cells including monocytes, endothelial cells, neutrophils, astrocytes, and mesenchymal cells among others (73). CXCL10 has affinity to CXCR3 and can regulate responses in T cells, natural killer cells, and monocytes (74).

A recent study supports a direct link between the CXCL10/CXCR3 axis and pain (27). After partial infraorbital nerve ligation injury, the TG of mice showed significantly higher levels of CXCR3 mRNA on days 3, 10, and 21 (when the experiment terminated); CXCR3 protein was also significantly upregulated on day 10 after injury as seen by WB. Furthermore, mRNA for the ligand CXCL10 was also upregulated on day 3 and 10 after injury, and both ligand and receptor were detected on TG neurons labelled with TUBIII (27) (Figure 1B). The higher chemokine axis levels associated with the injury suggests a pronociceptive effect of the chemokine and its receptor.

*In vivo* studies further support a direct role of the CXCL10/CXCR3 chemokine axis in pain (27). Intra-ganglionic injection of CXCL10 generated hypersensitivity, as measured by head withdrawal threshold. On the other hand, CXCR3 knockout mice showed lower hypersensitivity after CXCL10 injection. CXCL10 injection also resulted in increased ERK and AKT phosphorylation and no change in p38 and JNK phosphorylation, indicating that the pronociceptive effect of the CXCL10 axis could act through ERK and AKT signalling (27).

## Other Evidence of Chemokine Activation in Animal Models of Pain

Our literature search revealed other chemokines that were detected in animal models of trigeminal pain but were not the principal target of investigation. The following section details studies where nociception was assessed and chemokines were identified as a response to the injury, but a direct role (i.e., blocking or activating the chemokine axis) was not evaluated.

For instance, using a rat IONC model, a transcript analysis of several molecules from the TG identified overexpression of CCR2 at all time points tested (day 1, day 4, day 21) in comparison with the naïve group (12). In addition, in a transgenic mouse model overexpressing TNAα specifically in sensory neurons, CCL2 levels were significantly increased in the animal group associated with nocifensive behaviours as judged by reduced reward seeking (18).

In a rat model of nociception caused by mental nerve ligation, macrophage-1 antigen (MAC-1)-positive macrophages were found to co-express CCL3 in the injured nerve on days 3 and up to 28 post-injury, but this was not detected on day 88, when the experiment was terminated (22) (Figure 1A).

Furthermore, the role of chemokines in pain was evaluated in a study using TNFR1/R2 -/- KO mice in a “double hit” model of inflammation (38). In this model, mice were initially treated with CFA in the temporomandibular joint and then a second “hit” was given at a different site (colon). In both WT and KO mice, mechanical allodynia was present following the first “hit” but resolved by day 7 post CFA injection. By day 14, only the KO mice presented higher serum expression of CCL2, CCL5, CXCL9, and CXCL10, in contrast to the WT animals (mechanical allodynia was resolved in WT and KO animals by this time). After the second insult (21 days after the first), sensitivity for mechanical allodynia was higher only in the KO animals, suggesting that TNFR1/R2 -/- animals retained a level of residual sensitivity that could re-establish mechanical allodynia by a second insult at a different anatomical site (38).

Another type of trigeminal pain was studied by using a transgenic mouse model of familial hemiplegic migraine type 1 (FHM) generated by the knock in of the mutated form of Ca_v_2.1 Ca^2+^ channel (Cacna1a gene) (59). This study identified higher mRNA and protein expression of CCR2 (MCP-1) in mutant animals compared with WT animals as judged by RT-qPCR and WB (59), so suggesting a potential role for this chemokine in migraine.

A model of dry eye disease was used to study the role of transient receptor potential cation channel M8 (TRPM8) in ocular pain and inflammation in male mice (13). In this model, inhibition of TRPM8 with topical application of its antagonist M8-B (hydrochloride) significantly reduced the expression of CCL2 and CX3CR1 mRNA in the trigeminal nerve. M8-B also reduced corneal mechanical allodynia and eye closing ratio (pain signal) (13). The results allow us to speculate a role for CCL2 and CX3CR1 in trigeminal nociception, working downstream of TRPM8 (13).

CCL2 and CX3CR1 were also studied as part of the inflammatory response of a mouse ocular pain model of dry eye disease provoked by topical application of benzalkonium chloride (BAC) (15). BAC-treated animals manifested an increased nocifensive behaviour as measured by eye wiping and reduced weight gain when compared to control animals. CCL2 mRNA expression did not change in the trigeminal ganglion (CX3CR1 was not evaluated at this level). In the trigeminal Vc, CCL2 mRNA was significantly upregulated in the BAC-treated group, whereas CX3CR1 mRNA did not change. A causal role for the nocifensive behaviour given by the chemokines was not explored (15).

Bonfante et al. (34) investigated the acute (24 h) and persistent (7 and 14 days) hypernociceptive response to albumin-induced arthritis in the TMJ assessed by a formalin test in rats. Protein levels extracted from the Vc of animals in the persistent group showed significantly higher expression of P2X7, cathepsin S (CatS), and FKN, compared with the control group and the acute hypernociception group. The results suggest that the increased expression of FKN in the central nervous system (Vc) could be implicated in the maintenance of nociception. Interestingly, overexpression was not linked to microglial activation in Vc, as observed by the lack of significant change in protein expression of CD11b and p38MAPK (34). Whereas, in the spinal system, activation of the CatS/FKN pathway has been shown to be related to higher levels of CD11b and p38MAPK (75, 76), further studies are needed to investigate the cellular localisation of the components of the CatS/FKN/CX3CR1 pathway in Vc and to establish the participation of microglia in this pathway in Vc.

These results were validated in a separate study by the same group using a similar model of TMJ arthritis induced by CFA and methylated BSA (35). As expected, P2X7, FKN, and CatS levels were elevated in the trigeminal Vc. The chemokine response could be reversed by treatment with botulinum toxin type A (BoNT/A). In this study, microglial activation was not assessed; however, the authors propose that BoNT/A could be reducing P2X7 expression and therefore inhibiting the microglia–neuron crosstalk through CatS and FKN (35).

Somewhat contrasting evidence regarding FKN/CX3CR1 levels and orofacial nociception has been shown from a rat model of type 1 diabetes induced by streptozotocin (STZ) (36). In this model, nociception was evaluated by a count of reflex behaviour (ipsilateral face rubbing) during a repeated noxious stimulus with intra-TMJ capsaicin injection on days 7, 14, 21, and 28. The nociceptive response to capsaicin was significantly reduced on days 7, 14, 21, and 28 in diabetic animals in comparison with the control group, whereas CX3CR1 and pp38 protein expression in the trigeminal Vc of diabetic animals was upregulated at days 7 and 28 as seen by WB. FKN was also significantly higher in the diabetic trigeminal Vc only at day 7. The results indicate that in this model the FKN/CX3CR1 axis does not act as a pronociceptive factor at the trigeminal level in STZ rats (36). The contrasting findings from this model (showing increases in FKN/CX3CR1 with reduced nociceptive behaviour) compared to others (showing increases in expression of FKN/CX3CR1 linked with increased nociceptive responses) (34, 35) illustrates the complexity of nociceptive mechanisms in different pain conditions and indicates variability between different pain models. Interestingly, contrasting evidence from studies in the spinal system of STZ mice indicates that FKN/CX3CR1 could participate as a pronociceptive factor, as shown by paw withdrawal thresholds measured in KO and WT CX3CR1 STZ mice (77).

Evidence for a possible involvement of CXCL2 in pain comes from investigation of inflammatory pain in the rat at the level of the TG (32). CFA was injected into the masseter muscle to induce peripheral inflammation to investigate the role of transient receptor potential cation channel M2 (TRPM2) in inflammatory pain. *In vivo*, CXCL2 was not significantly upregulated 1 day after CFA injection as judged by RT-qPCR. However, TG cultures treated with ROS were able to trigger CXCL2 expression at transcript and protein levels as observed by RT-qPCR and ELISA. Interestingly, *in vitro* inhibition of either TRPM2 or peroxide reduced CXCL2 expression, suggesting a contribution of TRPM2 and ROS in the expression of CXCL2 in the TG (32).

Another study in male rats injected with CFA into the whisker pad showed CCL2 protein upregulation in the TG on days 1, 3, 7, and 14, resolving by day 21 when compared with the contralateral site (78). CFA-induced mechanical hyperalgesia was associated with P2Y14 receptor and CCL2 overexpression. Interestingly, inhibiting P2Y14 by intra-ganglionic injection of its antagonist attenuated the mechanical hyperalgesia and also reduced CCL2 protein expression in the TG. The results suggest that CCL2 may be acting downstream of the P2Y14 receptor in inflammatory pain (78).

Interestingly, there is also evidence of chemokines participating in anti-hyperalgesia (analgesic) roles. In a rat model of chronic orofacial pain with ligation injury of the masseter muscle tendon, Guo et al. (23) found that short-lived multipotent bone marrow stromal cells (BMSC) could have a long-term anti-hyperalgesia effect mediated by chemokines. The anti-hyperalgesia effect was dependent on CCL4 and CCR2 acting as inflammatory cell mediators, which lead to μ-opioid receptor (MOR) upregulation at the rostral ventromedial medulla (RVM) of the brainstem. The authors also found that activation of central μ-opioid receptors in this model was dependent on CXCL1/CXCR2 signalling, where CXCR2 is located in MOR-containing NeuN-positive neurons at the RVM (23) (Figure 1C). The authors also validated the role of CXCL1/CXCR2 in another model of nociception using CXCR2 knockout mice: upon IONC, CXCR2 knockout mice lacking the chemokine receptor did not show an anti-hyperalgesia (analgesic) effect upon BMSC injection (23).

In a study to investigate the effect of proinflammatory mediators on sensory neuron activity *in vitro*, dental pulp stem cells (DPSCs) primed with CGRP were used to collect conditioned medium and evaluate its effect in mouse TG cultures (33). Primed DPSCs were found to significantly overexpress CXCL1, CXCL8, CXCL14, CCL27, and CCL28. The neurons treated with primed DPSC conditioned medium had a greater calcium influx in response to capsaicin. Importantly, blockade of CXCR2 (CXCL1 and CXCL8 receptor) in the mouse TG cultures prevented the capsaicin response, suggesting that in this experiment, the chemokines released by the primed DPSC contributed to neuronal sensitization, particularly through CXCR2. Evidence of co-localization of CXCR2 with TRVP1 in the mouse TG neurons was used to propose a possible link and a novel pathway between these players to regulate neuron activity (33) (Figure 1B).

Jiang et al. (24) identified significant increases in GPR151 in the mouse TG after partial infraorbital nerve transection (pIONT) from day 3 until day 21 after injury. pIONT-induced mechanical allodynia was dependent on GRP151 as shown by comparing WT animals vs. GRP151 KO mice. After pIONT, GRP151 KO mice showed a reduced expression of the chemokines CCL5, CCL7, CXCL9, and CXCL10. Further observations showed that GRP151 acts through ERK signalling, which in turn positively modulates the chemokine expression in the TG of this model of nociception (24).

Overall, the link between injury and chemokine expression in different models allows a role for several chemokines in pain to be hypothesized. It would be interesting and valuable to continue to explore whether differential expression of these chemokines has a direct impact in the molecular mechanisms of nociception or whether their presence only relates to the inflammatory cascade in response to injury, without a causal role in pain.

## Link to Sexual Dimorphism in Pain Perception

Our search identified literature exploring sex-dependent differences in nociception and chemokine expression at the trigeminal level. A direct role of chemokines and pain was not concluded by any of the references in this section. However, the literature presented in this section invites further exploration of whether some chemokines may have a direct role in the sexual dimorphism of pain perception.

Some studies have shown an interesting link between pain and sex differences where chemokines may have a role. Kramer and Bellinger (79) found that castrated male rats subcutaneously injected with 17β-estradiol showed a significant antinociceptive effect in response to CFA injection into the TMJ. In relation to chemokine expression, CFA injection significantly increased the protein expression of CXCL2 and CCL20 in the TMJ, but the expression of both chemokines was independent of the estradiol level, indicating that expression of these chemokines is unrelated to the nociceptive changes observed by the addition of the steroid. This report provides a good example of how correlation between a chemokine expression level and pain behaviour does not necessarily show causality (79).

The effects of high and low plasma 17β-estradiol levels, together with intra-TMJ CFA or saline, on the expression of chemokine have also been studied in female rats (80). Comparison between high and low estradiol animals revealed that the profile of chemokine mRNA expression in TMJ, the trigeminal ganglion and Vc is modified by estradiol levels together with intra-TMJ CFA or saline injection. (Differences in CXC11, CCL25, CCL20, CCL21b, CCL4, CCL24, CCR3, and CCR4 expression were seen between high and low estradiol animals with intra-TMJ CFA, and in CXCL2, CCR5, CCR2,CCL2, and CCR7 between high and low estradiol animals with intra-TMJ saline). Whilst these findings indicate that the profile of chemokine expression in the trigeminal pathway can be modified by estradiol levels, this study did not evaluate differences between intra-TMJ CFA and saline, or any relationship of chemokine expression to nociceptive behaviours. However, a potential link with TMJ pain was suggested by the authors from observations made by others.

Additionally, there is evidence suggesting that the effect of inflammation on FKN/CX3CR1 expression in the TG could be sex dependent. Male rats injected with CFA or saline into the temporalis muscle showed significantly increased expression of CX3CR1 96 h post-injection compared with female rats (26). In addition, FKN expression in male rats was also significantly higher at 24 h post-injection (CFA or saline) when compared with female rats (26). Notably, the effect on CX3CR1 and FKN expression was also seen in the saline injection group (sham), suggesting a sex-dependent response to the injection and not the inflammatory substance in this experiment. However, a separate study involving CFA injection in the TMJ of male rats showed a significant upregulation of FKN-labelled cells in the TG when compared with control animals at day 14 (no comparison with female rats was made) (67). The data available suggests, at least partially, a possible sex-dependent difference of the role of this axis in pain modulation.

Overall, these reports support the notion that gonadal hormones could have a role in the sex differences of nociception modulated by chemokines. Nonetheless, a direct role for sex hormones differentially modulating chemokines and nociception in the trigeminal system is yet to be shown.

## Chemokine Presence in Human Cases of Trigeminal Pain

A number of reports have tried to elucidate a correlation between chemokines and pain in man. Particularly, we found studies associated with migraine and related headaches, tooth movement, osteoarthritis, pulpitis, and TMJ disorders. In some cases, there is a presence of chemokines in conditions that are associated with pain, but where the analysis did not explore a particular relation between them. In other cases, there are efforts to report a relation between pain and chemokine expression. In either case, it must be noted that a clear causal relationship cannot be concluded from any of the studies found:

Cerebrospinal fluid (CSF) from patients with migraine or tension-type headache contained significantly higher levels of CCL2 in comparison with healthy controls, suggesting a relationship between headache and CCL2 expression (41).

CCL2 in gingival crevicular fluid was found in a study investigating the biological response to orthodontic forces in adolescents and adults (42). Interestingly, a significant difference between group ages was observed upon the orthodontic tooth movement. In the younger group, CCL2 was elevated 1 day after the treatment started and resolved by day 7, whereas in the adult group CCL2 continued to be highly expressed at day 7 compared with the basal levels before treatment. Furthermore, the pain scores from each group followed a similar trend. The younger group experienced significantly higher pain scores only on day 1. On the contrary, by day 7, the adult group had a significantly higher pain score compared with the younger group. Nonetheless, it must be noted that the study design did not aim to correlate the levels of pain with the inflammatory response, and the trends described here are indirect observations (42). However, it can be observed that the adult population suffering from higher pain scores was also associated with a higher level of CCL2 expression in contrast to the younger group with lower pain scores.

Another report involving CCL2 studied synovial fluid from the TMJ of patients with internal derangement or osteoarthritis (40). Patients experiencing pain showed higher protein levels of CCL2 compared with patients without pain. Nonetheless, this difference was not significant and the sample number of patients with pain was not powerful enough to conclude a relation between chemokine expression and pain (40).

Acute trigeminal pain is observed in patients who undergo third molar extractions. Wang et al. (43) analysed tissue biopsies from these patients before and after tissue injury caused by the procedure. Pain scores were evaluated 3 h after the injury and analysed in relation to CCL2 transcription levels. CCL2 was positively correlated to the pain scores, supporting that CCL2 gene expression in the injured tissue may contribute to inflammatory pain (43).

A study of migraine patients revealed that CCL3 and CXCL8 were both found to be increased interictally in blood serum from patients with migraine in contrast to healthy control individuals (45). Additionally, blood analysis from migraine patients showed higher CXCL8 levels in samples collected 2 and 4 h post-attack onset (headache), in contrast to healthy controls (53). These results suggest that CXCL8 is overexpressed in patients suffering from migraine.

Similarly, CCL5 (RANTES) was found at significantly higher levels in blood serum from migraine patients during a headache attack, compared with healthy controls and with samples taken 1 week later from the same patients (48). Additionally, differential expression of CCL3 and CCL5 has been reported to be able to be used to distinguish between patients with migraine and those with tension-type headache (44).

Peripheral samples of necrotic jawbone from patients with atypical facial pain and trigeminal neuralgia caused by fatty-degenerative osteolysis and osteonecrosis of the jawbone (FDOJ) showed higher protein levels of CCL5 when compared with healthy jawbone tissue (47). There was no difference in other inflammatory markers such as IL-1ra, IL-6, IL-8, TNFα, and CCL2 between the groups. Removal of FDOJ tissue resulted in an overall 88% pain relief. Although the authors hypothesised a possible relation between pain relief and CCL5, it must be noted that such a relationship cannot be concluded from the data presented (47). This group also independently reported (46) that CCL5 could be detected in tissue removed from 301 patients with suspected bone marrow defects of the jawbone (categorized in seven disease groups), of which 47 belonged to the group “atypical facial and trigeminal pain.” No control group was evaluated and there were no differences between this group and other disease groups. The authors could make no conclusion in respect of a causal role for CCL5/RANTES in relation to pain in these patients (46). Nonetheless, it can be noted that at least in this cohort of 301 patients, CCL5 expression in the pain-related group was not different from other groups not linked to pain.

Other orofacial evidence suggesting a relationship of chemokines with pain in human comes from patients with acute pulpitis (50). In this study, patients who presented a high pain intensity, as measured by a verbal numeric scale (>5 on a 1–10 scale), showed significantly higher levels of CXCL8 (IL-8) protein in the gingival crevicular fluid of the affected tooth compared with the contralateral tooth. However, as no comparison was made between patients with high and low pain intensity, it is not clear whether the increase in CXCL8 was linked with the presence of pain or the presence of inflammation.

In this regard, CXCL8 is frequently found in studies related to trigeminal pain in patients and it is commonly reported as overexpressed as a result of the inflammatory response. For instance, patients suffering from irreversible pulpitis showed high CXCL8 expression in pulp tissue and pulp blood, but the chemokine was not significantly different in patients with higher pain scores (51, 52). Similarly, blood serum levels of CXCL8 in patients suffering from trigeminal neuralgia or hemifacial spasm were higher than those in healthy individuals, but the levels were not significantly correlated to pain scores (54).

Nonetheless, an interesting study investigated differences between partial and full responders to the analgesic ibuprofen in the reduction of pain after third molar extraction (81). The study found higher CXCL8 expression in the serum of full responders, and the authors proposed the inflammatory response as a variable in the analgesic efficacy. In this case, the relationship between CXCL8 and pain was not the study question, nonetheless, it provides an example of the multiple roles that chemokines can have surrounding nociceptive and antinociceptive mechanisms.

Finally, mutations affecting chemokine expression have also been linked to a lower propensity to suffer from headache. In this regard, the single nucleotide polymorphisms (V249I and T280M) affecting the expression of the chemokine receptor CX3CR1 have been associated with a lower risk of headache. Combadière et al. (49) reported that patients carrying either or both of the two CX3CR1 polymorphisms had a reduced risk of headache, suggesting that CX3CR1 might play a role in recurrent headache (49).

Overall, it can be noted that chemokines are present in patients experiencing conditions associated with trigeminal pain and in some cases, the expression levels seemed to correlate positively with pain scores. Nonetheless, the difficulty of distinguishing between an inflammatory response and a nociception role is also evident from these studies. Further research can benefit from more studies designed to correlate chemokine expression and quantitative pain scores in large-size populations.

## Conclusion

The present work aims to provide a summary of the current knowledge of chemokines in trigeminal pain. The evidence clearly exemplifies a pivotal role of some chemokines in pain modulation in different models of trigeminal pain (inflammatory, neuropathic, mechanical, and genetic).

The important role of chemokines in trigeminal nociception has been shown by chemokine or chemokine receptor inhibition, deletion or attenuation modifying pain behaviours triggered by a range of insults, highlighting their use as novel pharmacological targets. Further work is needed to dissect the underpinning mechanisms by which chemokines modulate pain sensation. The data gathered here suggest the participation of c-Fos, ERK, and p38 signalling cascades; the cation channels TRPM2 and TRPM8; as well as ROS and a possible role of sex hormones as key players in chemokine-driven pain modulation. Notably, with some exceptions (14, 22, 39), the specific role of macrophages/monocytes was overlooked. Future research on the trigeminal system could benefit from further exploration of the role of these cell types in the chemokine–chemokine receptor response to injury. It would also be interesting to compare whether chemokine responses are similar between trigeminal and spinal systems in specifically designed experiments. Investigating a particular chemokine activity at the same timepoints along the spinal and trigeminal tracts after an equivalent injury (e.g., nerve ligation, CFA injection) could help assess whether a potential treatment would have the same effect at both levels.

Finally, close attention to the timing should be considered in future research to understand when after the injury would be most effective to target a particular chemokine.

## Author Contributions

OS-C: preparation of draft manuscript, writing, reviewing, and editing manuscript. NW: assistance in drafting and reviewing manuscript. FB: writing, reviewing, and editing manuscript. All authors approved the final version of the article.

## Conflict of Interest

The authors declare that the research was conducted in the absence of any commercial or financial relationships that could be construed as a potential conflict of interest.

## Glossary

BSA, bovine serum albumin

CatS, cathepsin S

CCL, chemokine ligand

CCR, chemokine receptor

CFA, complete Freund's adjuvant

CGRP, calcitonin gene-related peptide

CSF, cerebrospinal fluid

CX3CR1, fractalkine receptor

CXCL, C-X-C motif chemokine ligand

CXCR, C-X-C motif chemokine receptor

DPSC, dental pulp stem cell

FKN, fractalkine

GFAP, glial fibrillary acidic protein

IAMNT, inferior alveolar and mental nerve transection

IB4, isolectin B4

IBA1, ionized calcium binding adaptor molecule 1

IHC, immunohistochemistry

IL, interleukin

IONC, infraorbital nerve constriction

IONL, infraorbital nerve ligation

KO, knockout

MAC-1, macrophage-1 antigen

NeuN, neuronal nuclear antigen

PAG, periaqueductal grey

pIONT, partial infraorbital nerve transection

RANTES, [the chemokine] regulated on activation, normal T expressed and secreted

ROS, reactive oxygen species

STZ, streptozotocin

TG, trigeminal ganglion

TMJ, temporomandibular joint

TNFα, tumour necrosis factor alpha

TNFR, tumour necrosis factor receptor

TRPA1, transient receptor potential cation channel, subfamily A, member 1

TRPM2, transient receptor potential cation channel, subfamily M, member 2

TRPM8, transient receptor potential cation channel, subfamily

M, member 8

TUBIII, class III β-tubulin

VGLUT2, vesicular glutamate transporter 2

WB, western blot

WT, wild type

XCL1, lymphotactin

XCR1, lymphotactin receptor.
